# iTRAQ-Based Quantitative Proteome Revealed Metabolic Changes in Winter Turnip Rape (*Brassica rapa* L.) under Cold Stress

**DOI:** 10.3390/ijms19113346

**Published:** 2018-10-26

**Authors:** Yaozhao Xu, Xiucun Zeng, Jian Wu, Fenqin Zhang, Caixia Li, Jinjin Jiang, Youping Wang, Wancang Sun

**Affiliations:** 1 College of Agronomy, Gansu Agricultural University, Lanzhou 730070, China; xuyaozhao@126.com; 2 College of Agronomy and Biotechnology, Key Laboratory of Hexi Corridor Resources Utilization of Gansu, Hexi University, Zhangye 734000, China; xiucunzeng@126.com (X.Z.); fenqinzh@hxu.edu.cn (F.Z.); pengli131@163.com (C.L.); 3 Jiangsu Provincial Key Laboratory of Crop Genetics and Physiology, Yangzhou University, Yangzhou 225009, China; wu_jian@yzu.edu.cn (J.W.); jjjiang@yzu.edu.cn (J.J.)

**Keywords:** *Brassica rapa*, turnip, differently accumulated proteins, cold stress

## Abstract

Winter turnip rape (*Brassica rapa* L.) is a large-scale winter-only oil crop cultivated in Northwest China. However, its cold-resistant molecular mechanism remains inadequate. Studying the cold adaptation mechanisms of winter turnip rape based on the proteomic technique of isobaric tags for relative and absolute quantification (iTRAQ) offers a solution to this problem. Under cold stress (−4 °C for eight hours), 51 and 94 differently accumulated proteins (DAPs) in Longyou 7 (cold-tolerant) and Tianyou 4 (cold-sensitive) were identified, respectively. These DAPs were classified into 38 gene ontology (GO) term categories, such as metabolic process, cellular process, catalytic activity, and binding. The 142 DAPs identified between the two cold-stressed cultivars were classified into 40 GO terms, including cellular process, metabolic process, cell, catalytic activity, and binding. Kyoto Encyclopedia of Genes and Genomes enrichment analysis indicated that the DAPs participated in 10 pathways. The abundance of most protein functions in ribosomes, carbon metabolism, photosynthesis, and energy metabolism including the citrate cycle, pentose phosphate pathway, and glyoxylate and dicarboxylate metabolism decreased, and the proteins that participate in photosynthesis–antenna and isoflavonoid biosynthesis increased in cold-stressed Longyou 7 compared with those in cold-stressed Tianyou 4. The expression pattern of genes encoding the 10 significant DAPs was consistent with the iTRAQ data. This study provides new information on the proteomic differences between the leaves of Longyou 7 and Tianyou 4 plants and explains the possible molecular mechanisms of cold-stress adaptation in *B. rapa*.

## 1. Introduction

Cold stress can lead to cell metabolic disorders, damage the cellular membrane system, and cause death. In order to survive, plants have developed complicated and effective cold-responsive mechanisms, such as variations in their leaf tissue structure, accumulation of compatible osmolytes, and the activation of cold-related genes [1,2,3,4]. Some plants can escape freezing injury by discarding their cold-sensitive structures, such as aboveground parts, or by shrinking vegetative organs into underground organs [5]. Cold stress can promote the expression of cold-related genes to improve tolerance tocold injury. Transcriptome analysis has identified many cold-related genes from diverse plants, furthering our understanding of cold stress [6,7,8,9]. However, proteins control the ultimate biological processes, and protein abundances depend on the regulation of transcription and post-transcription. Thus, the existence of an mRNA does not necessarily correspond to protein abundance and functional conformation [10,11]. Therefore, at the proteome level, understanding plant cold adaptation mechanisms is essential.

The proteomics technique is a systematic approach used to measure the entire protein abundance changes in specific biological situations and provides direct information about how cell metabolism is driven by proteins under stress response [12,13]. The isobaric tags for relative and absolute quantification (iTRAQ) technique is a high-throughput proteomic technique that allows simultaneous identification and quantification of proteins in multiple samples, with high coverage [14]. The iTRAQ technique is being applied for the study of the cold adaptation of plants [15,16,17,18,19,20].

Winter turnip rape (*Brassica rapa* L.) is a cold-tolerant and winter-only oil crop cultivated on a large scale in Northwest China [21]. In winter-turnip-rape-producing regions, long and chilly winters and relatively large differences in cold tolerance among varieties often result in overwintering failure and yield loss. Longyou 7 is the first cultivated variety of winter turnip rape with strong cold tolerance (more than 90% overwinter survival rate at −32 °C) [22]. Our recent research found that Longyou 7 has reduced photosynthesis, weaker growth, and earlier wilting of leaves than cold-sensitive varieties under cold stress [23,24]. We also identified some microRNAs (miRNAs) regulating the leaf senescence of cold-stressed Longyou 7 [25]; however, we lacked direct protein information about its cold tolerance. Therefore, analyzing the change pattern of its protein abundance under cold stress through a proteomic approach is important.

As the most sensitive organ for perceiving cold, leaves can respond rapidly to maintain functionality. This feature is crucial for winter survival and the reconstruction of all vegetative parts after the revival stage in the accumulation of photosynthates during cold acclimation [21,24,26]. Previous studies reported changes in proteomic profiles under cold stress in many plants, but proteomic studies of the leaves of winter turnip rape are lacking. In this study, iTRAQ technology was used to analyze proteome changes in response to cold stress in leaves of cold-tolerant Longyou 7 and cold-sensitive Tianyou 4 winter turnip rape varieties. We found that decreased photosynthesis, energy metabolism, and carbon, ribosome, and tryptophan metabolism are beneficial to the cold tolerance of winter turnip rape. Winter turnip rape can avoid cold injury by discarding their cold-sensitive structures, such as leaves and other above-ground organs. This study can serve as a basis for elucidating the possible molecular mechanism of cold tolerance in *B. rapa*.

## 2. Results

### 2.1. Analysis of Plant Growth and Physiological Indices under Cold Stress

**Figure 1:**
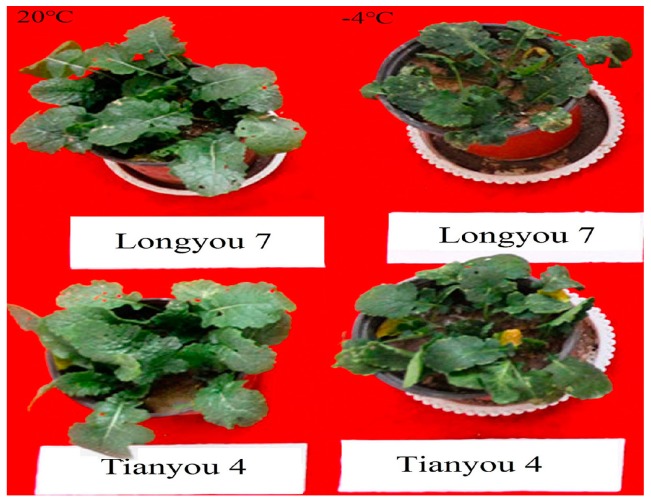
Effects of cold stress on winter turnip rape seedling growth.

**Figure 2:**
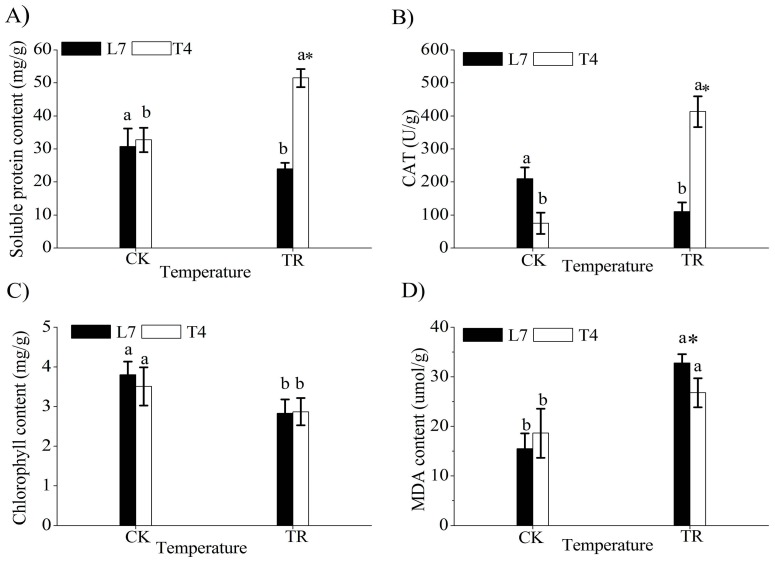
Responses of physiological indices under cold stress. (**A**) Soluble protein content. (**B**) Catalase (CAT). (**C**) Chlorophyll content. (**D**) Malondialdehyde (MDA). CK represents control treatment at 20 °C; TR refers to cold treatment at −4 °C; L7 and T4 represent cold-tolerant and cold-sensitive winter turnip rape varieties Longyou 7 and Tianyou 4, respectively; the mean and SD were calculated from three repeats of each treatment; bars show standard deviation; columns marked with different letters indicate significant statistical differences among different temperatures in the same variety based on Ducan’s multiple range tests (*p* < 0.05); the column marked with an asterisk was significantly different between L7 and T4 under TR treatment based on an independent-samples *t*-test (*p* < 0.05).

Cold stress resulted in leaf wilting and inhibited the aboveground growth of the two varieties, especially in Longyou 7 (Figure 1). Compared with the control (CK), soluble protein content and catalase (CAT) activity significantly increased in cold-stressed Tianyou 4 (T4TR) but decreased in cold-stressed Longyou 7 (L7TR) (Figure 2A,B). Under cold stress, chlorophyll content decreased in the two varieties (Figure 2C), but malondialdehyde (MDA) content increased (Figure 2D). Under cold stress (TR), the soluble protein content and CAT activity of Tianyou 4 (T4) were higher than those of Longyou 7 (L7) and the MDA content of the former was lower than that of the latter. There was no significant difference in chlorophyll content between the two varieties. These results indicated that the leaves of L7 have higher sensitivity to cold stress than do those of T4.

### 2.2. Protein Identification and Quantification

**Figure 3:**
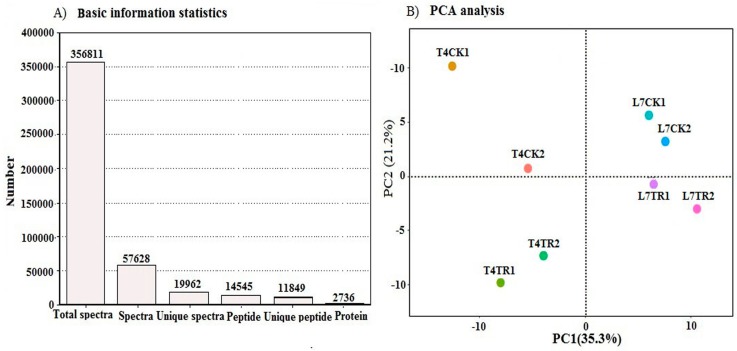
(**A**) Spectrum, peptides, and proteins identified from isobaric tags for relative and absolute quantification (iTRAQ) proteomics in winter turnip rape leaves. (**B**) Principle component analysis (PCA) of the proteome in two biological replicates from cold-stressed and non-stressed winter turnip rape leaves. L7CK and L7TR denote CK and TR treatments of the L7 variety, respectively. T4CK and T4TR denote CK and TR treatments of the T4 variety, respectively.

Leaf proteome profiles were obtained from winter turnip rapes by iTRAQ proteomics. A total of 356,811 spectra were generated from the leaves of the two winter turnip rape varieties and 57,628 spectra were matched to known spectra. Mascot identified 2736 proteins according to the threshold of 0.05 in the ions core cutoff (with 95% confidence) with false discovery rate (FDR) less than 1.04% and at least one unique peptide in each positive protein identification (Figure 3A and Table S1). The number of peptides and the distribution in length, mass, and sequence coverage of proteins of winter turnip rape leaves are provided in Figure S1. In addition, the results of the principal component analysis (PCA) showed that two biological replicates of L7CK, L7TR, and T4TR had good repeatability (Figure 3B). A further analysis of repeatability between two biological replicates of T4CK showed that their protein coverage was 89.08% based on a 50% variation (Figure S2), which was sufficient for the iTRAQ experiment according to a previous report [27]. These results demonstrated the reliability of the proteomics analyses.

### 2.3. Identification and Analysis of Differently Accumulated Proteins (DAPs)

**Figure 4:**
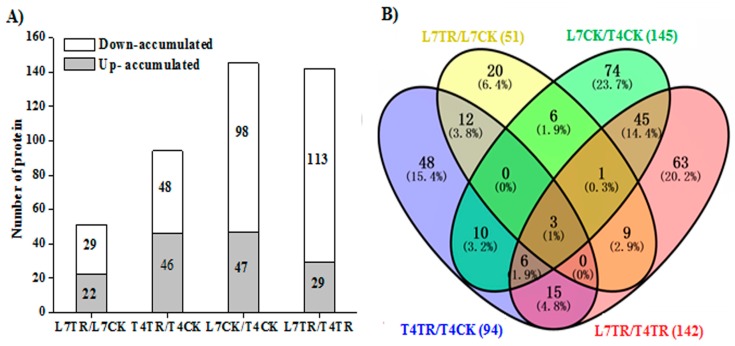
(**A**) Number of up- and down-accumulated differently accumulated proteins (DAPs) among different comparison groups. (**B**) Venn diagrams of DAPs identified by Isobaric tags for relative and absolute quantification (iTRAQ) among different comparison groups. L7TR/L7CK is the protein abundance ratio of L7TR compared to L7CK, T4TR/TR4CK is the protein abundance ratio of T4TR compared to T4CK, L7CK/T4CK is the protein abundance ratio of L7CK compared to T4CK, and L7TR/L4TR is the protein abundance ratio of L7TR compared to T4TR.

Significant DAPs were those whose fold change (FC) was greater than 1.5-fold or less than 0.67-fold and where *p* < 0.05. The DAPs of the two biological replicates in each sample were identified and a small number of DAPs were found among replicates, such as 1 DAP between L7CK1 and L7CK2, 9 DAPs between L7TR1 and L7TR1, 10 DAPs between T4CK1 and T4CK2, and 6 DAPs between T4TR1 and T4TR2 (Table S2); this agreed with the results of the PCA of the two biological replicates. Under cold stress, 51 DAPs were identified between L7TR and L7CK (Table S3), 94 DAPs were identified between T4TR and T4CK (Table S4), 145 DAPs were identified between L7CK and T4CK (Table S5), and 142 DAPs were identified between L7TR and T4TR (Table S6). Results showed that 22 (43%) proteins were more abundant and 29 (57%) were less abundant in L7TR/L7CK; 46 (49%) and 48 (51%) proteins were up- and down-accumulated in T4TR/T4CK, respectively; 47 (32%) and 98 (68%) DAPs were up- and down-accumulated in L7CK/T4CK, respectively; and 29 (20%) proteins were up- and 113 (80%) were down-accumulated in T4TR/L7TR (Figure 4A). Analysis of DAPs among different comparison groups showed that 15 DAPs were shared by L7TR/L7CK and T4TR/T4CK, implying that these common DAPs are stable in different cultivars under cold stress. There were 55 common DAPs in the L7CK/T4CK and L7TR/T4TR comparison groups (Figure 4B).

### 2.4. GO and KEGG Enrichment of DAPs under Cold Stress

**Figure 5:**
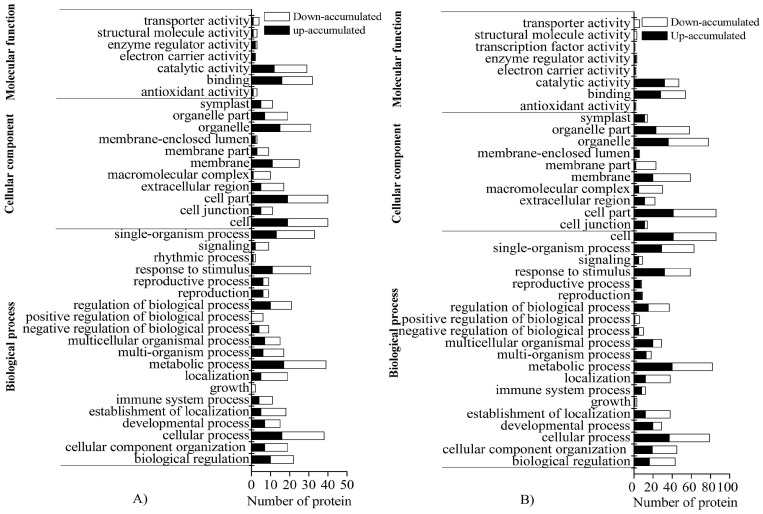
Gene ontology (GO) enrichment analysis of DAPs. (**A**) Comparison between L7TR and L7CK. (**B**) Comparison between T4TR and T4CK.

GO analysis revealed that the DAPs between L7TR and L7CK and those between T4TR and T4CK were classified into 38 functional terms (Figure 5A,B). For biological processes, the DAPs mainly participated in the metabolic, cellular, and single-organism processes and in response to stimulus. For cellular components, the DAPs were predominantly distributed in the cell, cell part, organelles, and membrane. For molecular function, the DAPs were mainly enriched in catalytic activity and binding.

**Figure 6:**
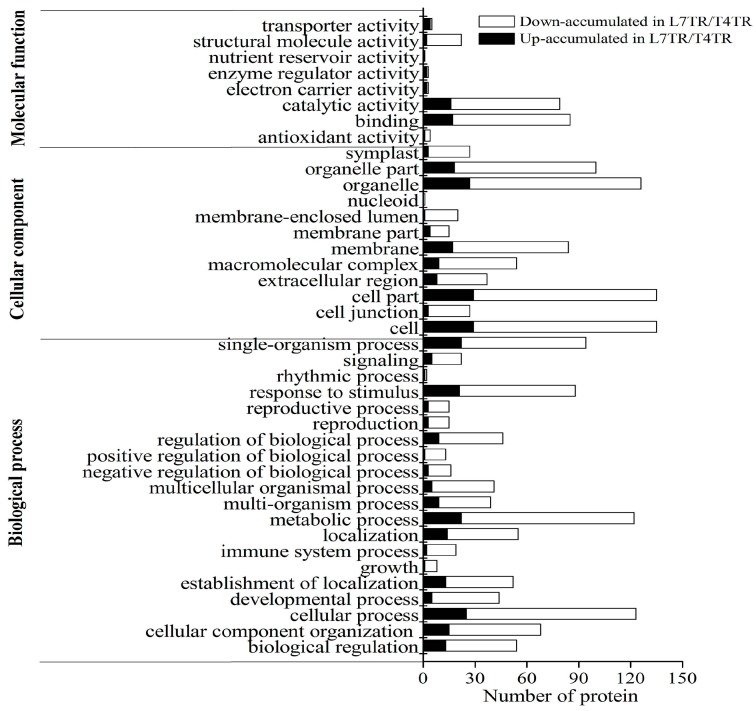
GO enrichment analysis of DAPs between L7TR and T4TR.

GO analysis revealed that the DAPs between L7TR and T4TR were classified into 40 GO terms. For the molecular function group, the most DAPs were responsible for catalytic activity and binding (Figure 6).

**Figure 7:**
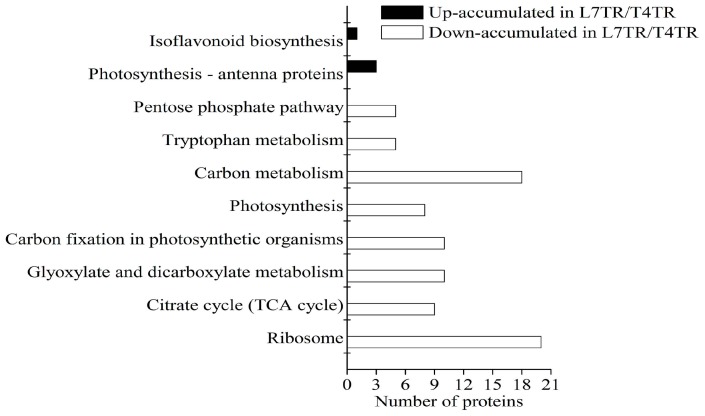
Kyoto Encyclopedia of Genes and Genomes (KEGG) pathway enrichment analysis of DAPs between L7TR and T4TR.

KEGG enrichment analysis displayed the pathways of DAPs between L7TR and T4TR (Figure 7 and Table S7). Only four up-accumulated DAPs in L7TR/T4TR were involved in photosynthesis–antenna proteins (path: ko00196) and isoflavonoid biosynthesis (path: ko03010). In addition, 85 down-accumulated DAPs in L7TR/T4TR were involved in ribosome (path: ko03010), citrate cycle (TCA cycle) (path: ko00020), glyoxylate and dicarboxylate metabolism (path: ko00630), carbon fixation in photosynthetic organisms (path: ko00710), photosynthesis (path: ko00195), carbon metabolism (path: ko01200), tryptophan metabolism (path: ko00380), and the pentose phosphate pathway (path: ko0003). Representative DAPs between L7TR and T4TR are summarized in Table 1 and detailed lists are provided in Tables S1 and S6.

### 2.5. Correlation between Protein Abundance and Gene Expression by qRT-PCR

**Figure 8:**
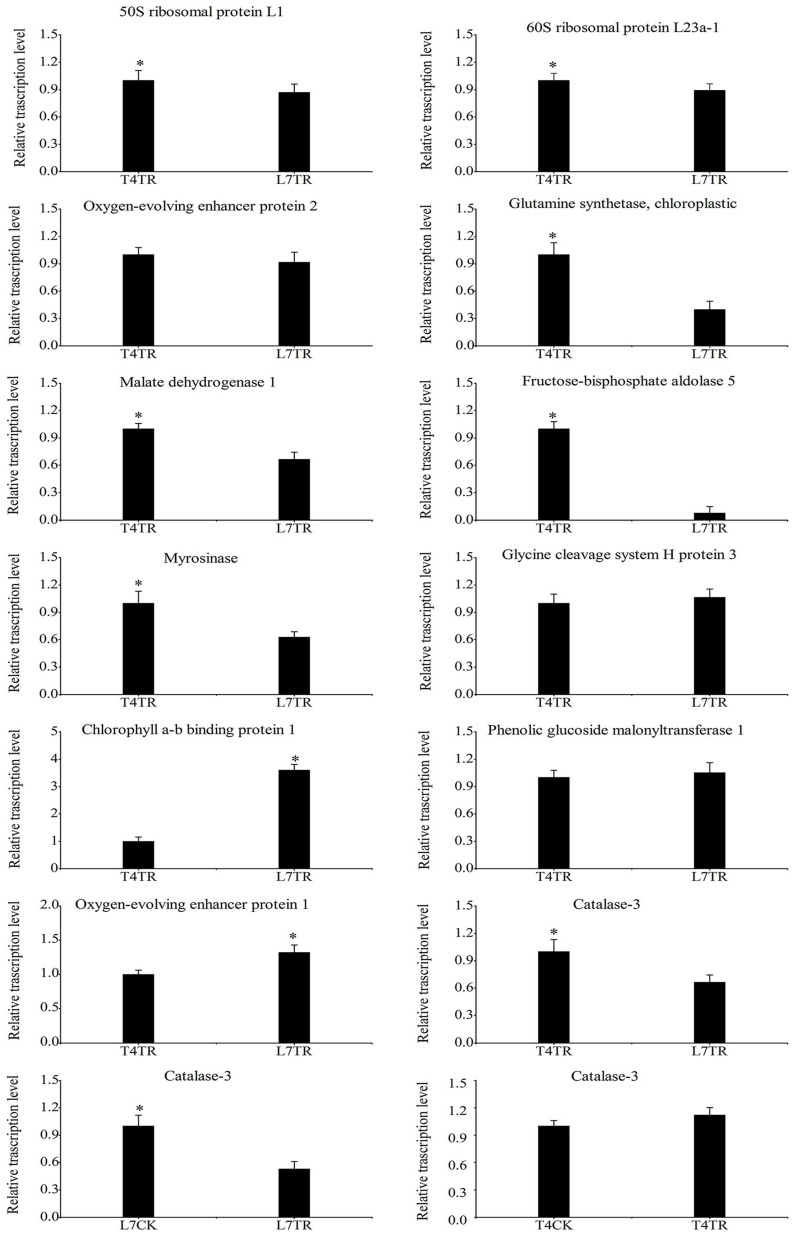
Analysis of DAP transcript levels by quantitative real-time polymerase chain reaction (qRT-PCR). Candidate genes were from differential protein genes between L7TR and T4TR. Statistically significant differences (Ducan’s multiple range tests *p* < 0.05) are indicated by asterisks.

To verify the correlation between protein abundance from iTRAQ analyses and their homologous gene expressions, a transcription analysis of 12 representative DAPs between T4TR and L7TR was performed by qRT-PCR (Figure 8). The results showed that the qRT-PCR data of 10 genes aligned with the iTRAQ results, as seen for 50S ribosomal protein L1, catalase-3, and myrosinase. Conversely, two genes that encoded oxygen-evolving enhancer protein 1 and glycine cleavage system H protein 3 showed the opposite tendency from their corresponding protein. In addition, transcriptional expression of catalase-3 between L7TR and L7CK as well as between T4TR and T4CK was analyzed. We found that the catalase-3 transcript was significantly down-regulated in L7TR compared with L7CK, and no significant changes in T4TR were observed compared with T4CK (Figure 8).

## 3. Discussion

Developmental inherent rhythm occurs in the cold tolerance development of plants. For example, winter crops show slower growth, lower-frequency cell division, and weaker metabolic activity after cold stress [28]. Vigorous growth negatively affects the cold tolerance of plants, whereas reduced growth activity improves this tolerance [23]. Overwintering plants can adapt and escape cold stress by leaf abscission and dormancy [29]. Some studies showed that a degree of leaf cold damage was not correlated with cold tolerance for winter wheat and barley, which was instead dependent on the crown acclimation ability [30,31,32]. In this study, leaves of cold-tolerant varieties suffered more serious cold damage than did those of cold-sensitive varieties (Figure 1). Therefore, the relation between degree of leaf cold damage and cold tolerance of varieties needs further study in winter rapes. Cold stress generally damages plant tissues due to the excessive production of reactive oxygen species (ROS), such as hydrogen peroxide (H_2_O_2_), which can cause lipid oxidation, destruction of membrane integrity, and metabolic and physiological disorders [33]. MDA is produced from lipid peroxidation, and the MDA level reflects damage to cell membranes [34,35]. CAT is the main H_2_O_2_-scavenging enzyme that helps plants to adapt to stress [36,37]. Plants respond to adversity by regulating the accumulation of osmotic substances, such as soluble proteins [38]. Therefore, MDA, CAT, and soluble proteins are usually used as vital physiological indices of stress responses.

The MDA content was higher in the leaves of cold-stressed Longyou 7 than in cold-stressed Tianyou 4, and CAT activity and soluble protein were lower in the former than in the latter, indicating that the aboveground parts of Longyou 7 experienced more severe cold damage than did those of Tianyou 4 (Figure 2). During winter, the aboveground tissue of winter turnip rape wilts, so the roots are critical for their survival in winter [24]. Thus, we speculated that the poor growth and premature senescence of the aboveground parts of Longyou 7, resulting from high sensitivity to cold-induced damage, decrease the nutrition consumption of roots and avoid cell freezing in the whole plant.

Previous research reported the molecular mechanisms of cold resistance in *Arabidopsis thaliana*, alfalfa, spinach, barley, and wheat [39]. However, the molecular mechanism underlying cold stress adaptation in winter turnip rape leaves remained unclear. Thus, proteomic analysis using iTRAQ was performed on leaves of two winter turnip rape varieties with differences in cold tolerance under cold stress and non-stressed conditions. After cold stress, 51 and 94 DAPs were identified in the leaves of Longyou 7 and Tianyou 4, respectively, and 142 DAPs were identified between the two cold-stressed varieties (Table S6). Further functional analysis revealed that some DAPs in the two varieties play an important role in some central metabolic pathways, which may be closely related to the response of winter turnip rape to cold stress.

### 3.1. Decreased Abundance of Photosynthesis-Related Proteins under Cold Stress

Photosynthesis is often the first process affected by stress given its sensitivity to abiotic stress [40]. In this study, most DAPs involved in photosynthesis were down-accumulated in cold-stressed Longyou 7 compared with in cold-stressed Tianyou 4 (Table S7), suggesting that photosynthesis in Longyou 7 was lower than that in Tianyou 4 under cold stress. This result agreed with previous reports [23]. Oxygen-evolving enhancer protein (OEE) is a chloroplast protein encoded by nuclear genes that plays essential roles in oxygen evolution and photosystem II (PSII) stability [41]. Its expression is considered a rate-determining step for the assembly of PSII subunits [42]. A previous study reported that OEE is involved in the cold acclimation in *Arabidopsis thaliana* [43] and *Triticum urartu* L. [44]. Under cold stress, the relative abundance of this protein was up-accumulated in leaves of cold-tolerant alfalfa [45] and winter barley [32]. However, our results showed that an abundance of OEE was down-accumulated in cold-tolerant Longyou 7 (L7TR) compared with cold-sensitive Tianyou 4 (T4TR) (Table 1). Therefore, further studies are needed to identify the role of OEE in response to cold stress in *B. rapa*. Reports showed that light energy capturing and charge-separation are largely irrelevant for temperature during photosynthesis [46]. Chlorophyll a,b binding protein was up-accumulated in cold-stressed Longyou 7 compared with in cold-stressed Tianyou 4 (Table 1). The down-accumulated chlorophyll a,b binding proteins and OEE may mitigate the photodamage caused by increasing ROS formation due to the over-energized state of the thylakoid membrane [17]. Overall, we speculate that the severe inhibition of photosynthesis in the leaves of Longyou 7 maybe because the down-accumulated OEEs did not match the up-accumulated chlorophyll a,b binding proteins in the cold-stressed leaves of Longyou 7. In addition, a transcription analysis of oxygen-evolving enhancer protein 1 was performed by qRT-PCR, and we found that the expression of its gene showed the opposite pattern to the protein levels (Figure 8); this might be due to post-transcriptional regulation, such as mRNA turnover, translation rate, and/or post-translational protein stability.

### 3.2. Decreased Abundance of Energy-Metabolism-Related Proteins under Cold Stress

Respiration is the center of material and energy metabolism; it can generate chemical energy, reduce power, and produce material for the synthesis of other important organic components in plants through complex biochemical steps [47]. In this study, the citrate cycle (TCA cycle) and pentose phosphate pathway (PPP) were inhibited more in the leaves of cold-stressed Longyou 7 than in cold-stressed Tianyou 4, accompanying the down-accumulation of cytosolic isocitrate dehydrogenase [NADP](NADP-ICDH), aconitate hydratase, mitochondrial, succinate-CoA ligase (ADP-forming) subunit beta, malate dehydrogenase (MDH), transketolase (TKT), and ribulose-phosphate 3-epimerase (Table 1 and Table S7). The results align with the suggestion that decreased respiration is conducive to sugar accumulation and increased cold tolerance [48,49].

Glyoxylate and dicarboxylate metabolism play vital roles in balancing metabolic disorders of plants and transporting energy to strengthen stress tolerance under stress conditions [50]. In this study, glyoxylate- and dicarboxylate-metabolism-related proteins were down-accumulated more in cold-stressed Longyou 7 than in cold-stressed Tianyou 4 (Table S7). Of these proteins, glutamine synthetase (GS) and glycine cleavage system H protein (GCSH) are important proteins related to photorespiration [51,52]. GS is the rate-limiting enzyme for re-assimilation of ammonia in photorespiration [53,54], and overexpression of its gene enhances photorespiration capacity and tolerance to adverse stress [50,55]. GCSH is a small lipoylated protein that enhances the activity of the glycine cleavage system [39]. Overexpression of the GCSH gene resulted in an increase in photosynthesis and biomass in *Arabidopsis thaliana* [56]. A previous study reported that the abundance of GS in cold-tolerant rhododendrons was higher than in cold-sensitive varieties [57]. A study on rice anthers found that GCSH was up-accumulated in the cold-sensitive variety, but did not significantly change in the cold-tolerant cultivar after cold stress [58]. However, in our study, cold-tolerant Longyou 7 had less GS and GCSH compared with cold-sensitive Tianyou 4 (Table 1). Further analysis should be conducted on GS and GCSH in the future. We also found that the changing trends between GCSH protein abundance and its gene expression were not consistent (Figure 8 and Table 1), which might be related to post-transcriptional regulation and/or post-translational protein stability.

### 3.3. Decreased Abundance of Carbon-Metabolism-Related Proteins under Cold Stress

Compared with cold-stressed Tianyou 4, all the DAPs associated with carbon metabolism and carbon fixation in photosynthesis were down-accumulated more in cold-stressed Longyou 7 than in cold-stressed Tianyou 4. Fructose-bisphosphate aldolase (FBPA) was reduced considerably (Table 1). FBPA is a vital enzyme involved in the glycolytic/gluconeogenic pathway, and it plays important roles in abiotic stress responses [59]. Under chilling stress, FBPA is also down-accumulated in soybean and winter wheat [60,61]. Catalase-3 (CAT3) is the key enzyme that scavenges hydrogen peroxide generated through respiration in the mitochondria [62,63,64]. It enhances the chilling resistance of plants [65]. CAT3 was down-accumulated in cold-stressed Longyou 7 leaves compared with in cold-stressed Tianyou 4 leaves (Table 1), and this result was consistent with the decline in CAT activity (Figure 2). CAT has three isozymes with biochemical differences—CAT1, CAT2, and CAT3—encoded by three unlinked catalase genes, *Cat1*, *Cat2*, and *Cat3*, respectively. The three genes have differences in their temporal spatial expression [66,67]. In the present study, we found that the CAT activity of Tianyou 4 was significantly higher under cold stress than under its control (Figure 2). However, we did not identify any of this enzyme-related protein via iTRAQ analysis. We speculated that high CAT activity is the result of the accumulation of three catalase isozymes (CAT1, CAT2, and CAT3), but any single *Cat* gene product has no significant difference between cold- and non-stressed Tianyou 4. Further analysis also showed that catalase-3 (*Cat3*) expression at the transcriptional level was not significantly different between cold-stressed (T4TR) and non-stressed Tianyou 4 (T4CK) (Figure 8), which was consistent with the result of the proteomic analysis. MDH is an important enzyme involved in multiple metabolic pathways, such as the TCA cycle, photosynthetic metabolism, and glyoxysomes, and can catalyze the interconversion of oxaloacetate and malate [68]. Previous studies reported that MDH overexpression in plants could alter some biological processes, such as hormone signal transduction, and increase plants’ cold tolerance along with decreasing ROS levels [69,70]. In the present study, four MDH-related DAPs (two cytoplasmic MDH and two glyoxysomal MDH) were significantly down-accumulated in the leaves of Longyou 7 compared with in those of Tianyou 4 under cold stress (Table 1). This result suggests that the leaves of Longyou 7 are more vulnerable to cold damage than those of Tianyou 4.

### 3.4. Decreased Abundance of Ribosome-Related Proteins under Cold Stress

Protein biosynthesis is influenced by ribosome assembly and efficient use [71]. The up-accumulated ribosomal proteins help plants to resist adverse stress [15,72]. In the present study, the DAPs (50S ribosomal proteins and 60S ribosomal proteins) participating in the ribosome were down-accumulated more in leaves of Longyou 7 than in the leaves of Tianyou 4 after cold stress (Table 1 and Table S7), implying that cold stress inhibited the protein synthesis of the leaf cells of Longyou 7.

### 3.5. Other Metabolism-Related Proteins under Cold Stress

Myrosinase (MYR) is a glucosinolate hydrolase that degrades glucosinolates and produces potent biological activities for plant stress defense and plant growth regulation [73]. In this study, four DAPs associated with MYR participating in tryptophan metabolism were identified, and these DAPs were down-accumulated in the leaves of Longyou 7 compared with in those of Tianyou 4 after cold stress (Table S7). These results suggest that the cold tolerance and growth in the leaves of Longyou 7 are weaker than in the leaves of Tianyou 4.

Isoflavonoids are plant-derived heterocyclic phenolic secondary metabolites with antioxidant activities [74]. Under cold stress, an increase in isoflavonoid levels can help protect plant organs against cold injury [75]. Phenolic glucoside malonyltransferase (PMAT) is the key enzyme in plant isoflavone biosynthesis, and an increase in its activity can accelerate isoflavonoid anabolism and enhance plant antioxidant abilities [76,77]. In our study, PMAT1 was more up-accumulated in the leaves of Longyou 7 than in those of Tianyou 4 after cold stress (Table 1).

Together with the decline in photosynthesis, respiration, carbon metabolism, ribosome metabolism, and tryptophan metabolism effectively weaken aboveground growth and accelerate early leaf senescence in Longyou 7; these decrease the nutrient consumption in the roots and avoid cell freezing in the entire plant. We concluded that winter turnip rape features a cold-responsive mechanism for avoiding further cold injury by discarding cold-sensitive structures such as leaves and other aboveground organs.

## 4. Materials and Methods

### 4.1. Plant Materials and Cold Stress Treatment

Two winter turnip rape (*Brassica rapa* subsp. *rapa*) cultivars, Longyou 7 (L7, cold-tolerant, with a more than 90% overwinter survival rate at −32 °C) and Tianyou 4 (T4, cold-sensitive, with a 62.4% overwinter survival rate at −9 °C), were used as experimental materials. The seeds were provided by the Rapeseed Engineering Research Center, Gansu Agricultural University (Lanzhou, China). Winter turnip rapeseeds were planted in a plastic pot (18 cm diameter, 15 cm deep) filled with a mixture of garden soil and sand (3:1, *w*/*w*) in a greenhouse at 20 °C with a 16/8 h (light/dark) cycle and photosynthetic active radiation (PAR) of 450 μmol·m^−2^·s^−1^ until the six-leaf stage, then transferred into an artificial climate (Safu, Ningbo, China) for cold treatment following a previously described method [25]. The temperature was decreased at a rate of 2 °C·h^−1^ and then held at 10 °C for 48 h, at 4 °C for 48 h, and at −4 °C for 8 h. The plants grown at −4 °C were used as cold treatments (TR) and the untreated plants (20 °C) were used as controls (CK). The leaves at −4 °C (TR) and 20 °C (CK) were collected, quick-frozen in liquid nitrogen, and stored at −80 °C for further analysis. The samples were named T4CK (leaf of Tianyou 4 at 20 °C), T4TR (leaf of Tianyou 4 treated at −4 °C), L7CK (leaf of Longyou 7 at 20 °C), and L7TR (leaf of Longyou 7 treated at −4 °C). Leaf tissue from every five plants was pooled as one biological replicate, and two biological replicates of each treatment were used for the isobaric tags for relative and absolute quantification (iTRAQ) analysis.

### 4.2. Analysis of Physiological and Biochemical Indices

Soluble protein content and catalase (CAT) activity were analyzed according to the methods described by Bradford [78] and Cakmak et al. [79], respectively. Chlorophyll and malondialdehyde (MDA) contents were measured as described by Arnon [80] and Campos et al. [81], respectively. These indices were determined on a U-3900H ultraviolet–visible spectrophotometer (Hitachi Limited, Tokyo, Japan). Leaf tissue from every five plants composed one biological replicate and each treatment included three biological replicates for the analysis of physiological indices.

### 4.3. Protein Extraction and iTRAQ Labeling

Total proteins from each sample were extracted following the method described by Yang et al. [82]. Each sample included two biological replicates. Concentrations and the quality of proteins were determined according to Bradford [78] and sodium dodecyl sulfate polyacrylamide gel electrophoresis (SDS-PAGE), respectively. Equal-quality proteins from each sample were used for iTRAQ analysis at the Beijing Genomics Institute (BGI, Shenzhen, China). The samples were digested with trypsin (Promega, Madison, WI, USA) for 16 h at 37 °C and at a trypsin/protein ratio of 1:20. Their constituted peptides were combined with 0.5 M triethylammonium bicarbonate (TEAB) and processed following the manufacturer’s protocol for 8-plex iTRAQ reagent labeling (Applied Biosystems, Foster City, CA, USA). In detail, the leaf samples of Tianyou 4 were labeled with iTRAQ tags 113/114 (CK) and 115/116 (cold treatment). The leaf samples of Longyou 7 were labeled with tags 117/118 (CK) and 119/121 (cold treatment). All labeled samples were then mixed and vacuum-dried.

### 4.4. Separation of Peptides and LC–MS/MS Analysis

Mixtures of iTRAQ-labeled peptide were fractionated on an LC-20AB HPLC system (Shimadzu, Kyoto, Japan) by strong cationic exchange, as discussed previously [83]. Finally, 20 filtered fractions were collected, desalted with a Strata X C18 column (Phenomenex, Torrance, CA, USA), and vacuum-dried [84,85]. Each dried fraction was resolved in a buffer (5% acetonitrile and 0.1% formic acid). After centrifugation at 20,000× *g* for 10 min, the supernatant was adjusted to a final concentration of 0.5 μg/μL. Next, 5 μL of peptide was loaded on a LC-20AD Nano-HPLC system (Shimadzu, Kyoto, Japan) with a 2 cm C18 trap column (inner diameter, 200 μm), and then eluted onto an analytical C18 column (inner diameter, 75 μm) packed in-house.

The liquid chromatography–electrospray ionization–tandem mass spectrometry (LC–ESI–MS/MS) of the fractionated samples was performed as previously described [82] on a Triple TOF 5600 System (AB SCIEX, Concord, ON, Canada) at BGI (Shenzhen, China). Data were acquired with an ion spray voltage of 2500 V, 30 psi nitrogen pressure, and an interface heater temperature of 150 °C. For information-dependent data acquisition, survey scans were acquired every 250 ms, and as many as 30 production scans exceeding a threshold of 120 counts per second (counts/s) with a 2+ to 5+ charge state were collected [86]. Sweeping collision energy and dynamic exclusion were set to 35 eV and half of the peak width (15 s), respectively [87].

### 4.5. iTRAQ Protein Identification and Quantification

The Mascot 2.3.02 search engine (Matrix Science, London, UK) was used for iTRAQ protein identification and quantification. The *B. rapa* protein database 2.0 [88] containing 45,270 sequences was selected as the analytical database. The parameters for protein identification were used as described previously [86,89,90]. The mass-spectrometry-based proteomics data are available via ProteomeXchange with the identifier PXD008195. Protein ratios were quantified and normalized by the median ratio in Mascot. Differentially accumulated proteins (DAPs) were determined by permutation test analysis using Bonferroni multiple testing correction. Proteins with a >1.5-fold or <0.67-fold change between the two samples and a *p*-value less than 0.05 were considered significant.

The functional categorizingof the DAPs was performed using gene ontology (GO, http://geneontology.org/). The metabolic pathway of DAPs was predicted using the Kyoto Encyclopedia of Genes and Genomes (KEGG) (http://www.genome.jp/kegg/) database. A *p*-value ≤ 0.05 was used as the threshold of significant enrichment of GO and KEGG pathways.

### 4.6. RNA Extraction and qPCR Analysis of Gene Expression

The total RNA of leaves from the two varieties at −4 °C (TR) and 20 °C (CK) was isolated using TRNzol Universal Reagent (Tiangen Biotech CO., Beijing, China) in accordance with the manufacturer’s instructions. A SuperScript^®^III RT Reagent Kit (Invitrogen, Beijing, China) was used for the synthesis of first-strand cDNA. Quantitative real-time polymerase chain reaction (qRT-PCR) was performed on a 7900HT Fast Real-Time PCR System (Applied Biosystems, Foster City, CA, USA) with Synergy Brands (SYBR) qPCR Mix (Invitrogen, Shanghai, China). The amplification procedure followed that in a previous report [25]. The primer sequences of the corresponding protein genes are listed in Table S8, and the *actin* of *B. rapa* was used as the internal standard. Three biological replicates and three technical replicates were performed for each gene. The relative expression of genes was analyzed using the 2^−ΔΔ*C*t^ method [91].

## 5. Conclusions

In this study, iTRAQ was used to study the differential proteomics of winter turnip rape leaves under cold stress, and 51 and 94 DAPs in Longyou 7 and Tianyou 4 were identified, respectively. In addition, 142 DAPs were identified between the two cold-stressed varieties. Based on the functional analysis, we concluded that decreased energy metabolism via the TCA cycle, PPP, glyoxylate, and dicarboxylate, together with decreased photosynthesis, enabled winter turnip rape to balance synthesis and consumption of sugar. This adaptive balance permitted the survival of winter turnip rape under cold stress. Moreover, the decreased metabolism of carbon, ribosomes, and tryptophan can be considered a reason for the enhancement of the cold tolerance of winter turnip rape by decreasing the growth activity of its aboveground parts. In summary, these findings help elucidate the molecular mechanisms involved in the cold tolerance of plants.

## Figures and Tables

**Table 1:** Representative differentially accumulated proteins (DAPs) in two cold-stressed varieties of winter turnip rape.

Accession (Uniprot)	Uniprot_Swissprot Description	Fold Changes ^a^		
		L7TR/L7CK	T4TR/T4CK	L7TR/T4TR
**Ribosome**				
Q9LY66	50S ribosomal protein L1	-	-	0.348519904
Q9SKX4	50S ribosomal protein L3-1	-	0.636134306	0.365801714
Q9SLF7	60S acidic ribosomal protein P2-2	-	-	0.483258248
Q8LD46	60S ribosomal protein L23a-1	-	-	0.352601546
P36210	50S ribosomal protein L12-1	-	-	0.364051915
P92959	50S ribosomal protein L24	-	-	0.366241266
**Citrate cycle (TCA cycle)**				
Q9SRZ6	Cytosolic isocitrate dehydrogenase [NADP]	-	1.757858283	0.611169392
Q9SIB9	Aconitate hydratase 3	-	-	0.659753955
O82662	Succinate-CoA ligase [ADP-forming]	-	-	0.633851199
**Glyoxylate and dicarboxylate metabolism**				
Q42624	Glutamine synthetase	-	-	0.36059385
Q9LQL0	Glycine cleavage system H protein 3	-	-	0.388785817
**Carbon fixation in photosynthetic organisms**				
Q8RWV0	Transketolase-1	-	1.696959938	0.611169392
Q9ZTP5	Ribulose-phosphate 3-epimerase	-	-	0.597820343
**Photosynthesis**				
Q96334	Oxygen-evolving enhancer protein 2	-	-	0.449814881
P83504	Oxygen-evolving enhancer protein 1	-	-	0.375329761
Q8W0Y8	Photosystem II reaction center PSB28	-	-	0.405032816
**Carbon metabolism**				
Q42547	Catalase-3	0.568893536	-	0.550541856
P93819	Malate dehydrogenase 1, cytoplasmic	-	-	0.595674747
Q9LF98	Fructose-bisphosphate aldolase 8	-	-	0.405152525
P25697	Phosphoribulokinase, chloroplastic	-	-	0.552468889
Q43743	Malate dehydrogenase 1, glyoxysomal	-	1.672743344	0.579876978
O65581	Fructose-bisphosphate aldolase 5	-	-	0.348710843
**Tryptophan metabolism**				
Q00326	Myrosinase	0.518096445	-	0.495250588
**Pentose phosphate pathway**				
Q8RWV0	Transketolase-1, chloroplastic	-	-	0.611169392
Q43157	Ribulose-phosphate 3-epimerase	-	-	0.553787078
**Photosynthesis—antenna proteins**				
P27525	Chlorophyll a-b binding protein CP24	-	0.574349177	1.601593552
P04778	Chlorophyll a-b binding protein 1	-	-	1.818756629
**Isoflavonoid biosynthesis**				
Q940Z5	Phenolic glucosidemalonyl transferase 1	-	-	1.519359178

^a^ Fold changes, - means no differential accumulation detected.

## Supplementary Materials

The following are available online at http://www.mdpi.com/1422-0067/19/11/3346/s1, Table S1: Overview of proteins identified in winter turnip rape under cold stress, Table S2: Differentially accumulated proteins between two biological replicates of each sample in L7 and T4 leaves under cold-stressed and non-stressed conditions, Table S3: Differentially accumulated proteins between L7TR and L7CK, Table S4: Differentially accumulated proteins between T4TR and T4CK, Table S5: Differentially accumulated proteins between L7CK and T4CK, Table S6: Differentially accumulated proteins between L7TR and T4TR, Table S7: KEGGpathway enrichment analysis of DAPs between L7TR and T4TR, Table S8: Primers for qRT-PCR analysis of DAPs genes, Figure S1: (A) The length and (B) number distribution of peptides, (C) sequence coverage, and (D) mass of proteins, Figure S2: The repeatability of two biological replicates of each sample.

## Abbreviations

iTRAQ	Isobaric tags for relative and absolute quantification
2DE	Two-dimensional electrophoresis
CAT	Catalase
MDA	Malondialdehyde
PCA	Principal component analysis
TEAB	Triethylamonium bicarbonate
CK	Control
DAPs	Differently accumulated proteins
GO	Gene ontology
KEGG	Kyoto Encyclopedia of Genes and Genomes
qRT-PCR	Quantitative real-time polymerase chain reaction
ROS	Reactive oxygen species
OEE	Oxygen-evolving enhancer protein
PSII	Photosystem II
PSI	Photosystem I
TCA	Citrate cycle
PPP	Pentose phosphate pathway
NADP-ICDH	Accompanying cytosolic isocitrate dehydrogenase (NADP)
ACO	Aconitate hydratase, mitochondrial
SUCLA	Succinate-CoA ligase (ADP-forming)subunit beta
MDH	Malatedehydrogenase
TKT	Transketolase
FBPA	Fructose-bisphosphate aldolase
RPE	Ribulose-phosphate 3-epimerase
GS	Glutamine synthetase
GCSH	Glycine cleavage system H protein
GCS	Glycine cleavage system
CAT3	Catalase3
MDH	Malate dehydrogenase
OAA	Oxaloacetate
MYR	Myrosinase
PMAT	Phenolic glucosidemalonyl transferase
