# Supplementary figures and images for: iTRAQ-Based Quantitative Proteome Revealed Metabolic Changes in Winter Turnip Rape (Brassica rapa L.) under Cold Stress
Source: Int J Mol Sci. 2018 Oct 26;19(11):3346. doi: 10.3390/ijms19113346 (PMC6274765; doi:10.3390/ijms19113346)

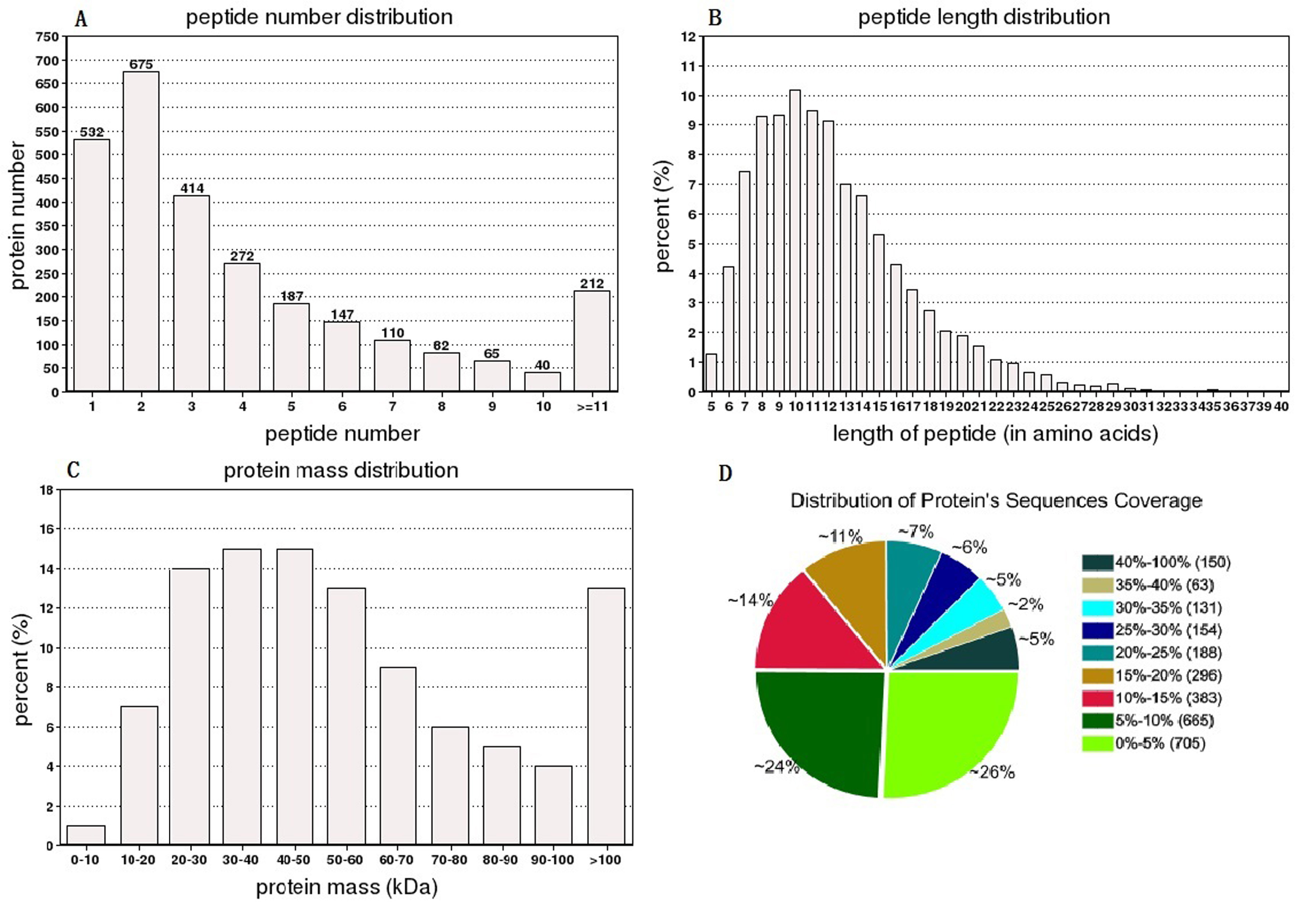

Supplement: Supplementary file 1 [file ijms-19-03346-s001.zip › supplementary/Figure S1.jpg]

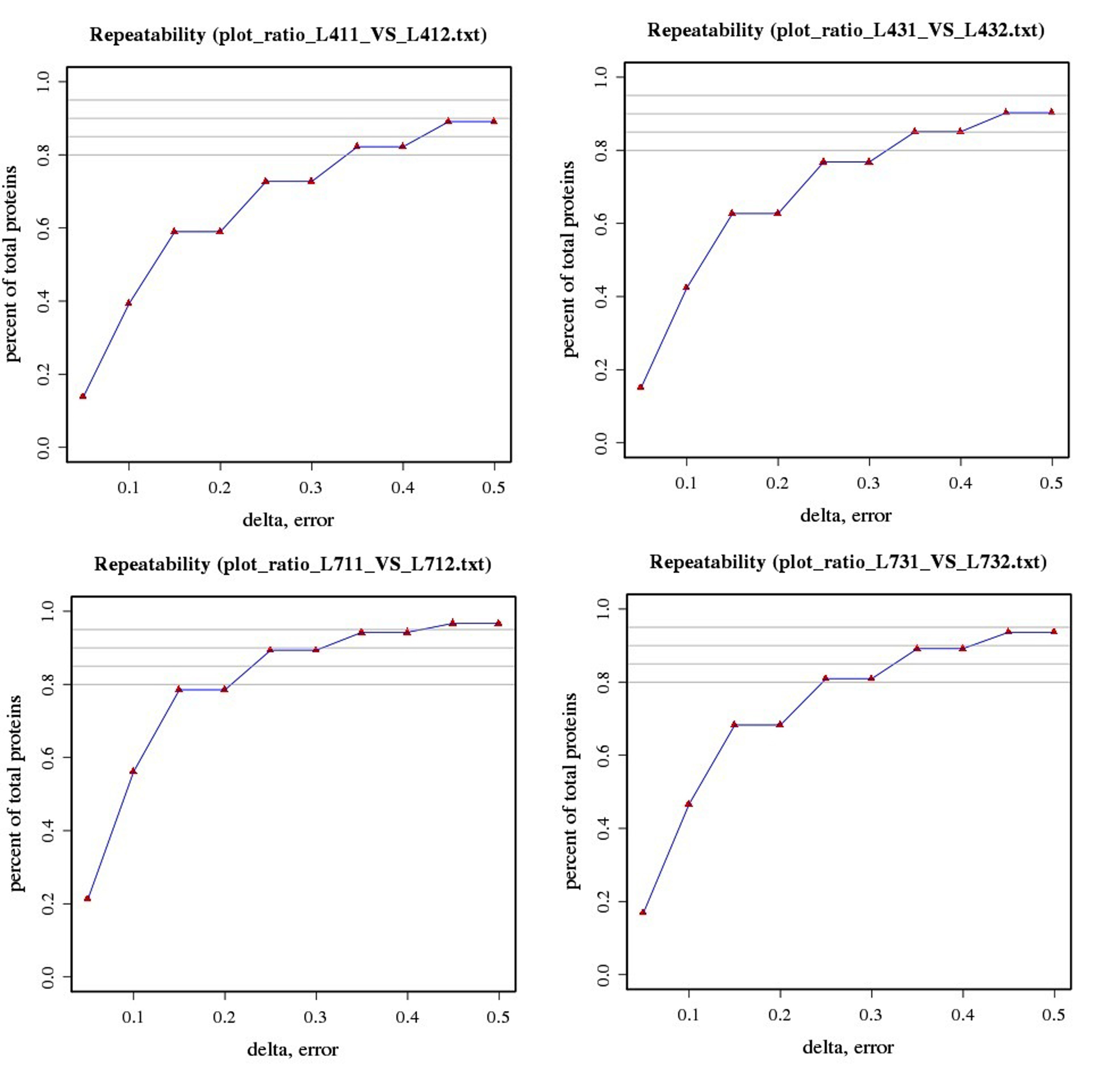

Supplement: Supplementary file 1 [file ijms-19-03346-s001.zip › supplementary/Figure S2.jpg]
