# Supplementary material for: iTRAQ-Based Quantitative Proteome Revealed Metabolic Changes in Winter Turnip Rape (Brassica rapa L.) under Cold Stress
Source: Int J Mol Sci. 2018 Oct 26;19(11):3346. doi: 10.3390/ijms19113346 (PMC6274765; doi:10.3390/ijms19113346)
Supplement: Supplementary file 1 [file ijms-19-03346-s001.zip › supplementary/Table S8.doc]

**Table S8 Primers for qRT-PCR analysis of genes of differentially expressed proteins**

| **Uniprot ID** | **Protein name** | **Primer Sequences (5'--3')** | |
| --- | --- | --- | --- |
|  |  | **primer F** | **primer R** |
| Q9LY66 | 50S ribosomal protein L1, chloroplastic | GTAAACCAACCAGCAATG | TTCGCTTAGTCCTCTTCTC |
| Q8LD46 | 60S ribosomal protein L23a-1 | CAAACAGACTCCGAGAAAG | CTCAAGAATCCCAACAACT |
| Q8W0Y8 | Oxygen-evolving enhancer protein 2 | CTTCGGGAGGCATTCACC | TCAATTCCAAGCGAGACC |
| P83504 | Oxygen-evolving enhancer protein 1 | AATCCAGCAGCGGTTATG | TCACAGGTCCAAGCCAAT |
| Q42547 | Catalase-3 | ACAGCAGGGATGTTGATA | AGGATGGCTAAGATGAGA |
| P93819 | Malate dehydrogenase 1, cytoplasmic | CTAATAGTGGGTCAGGGTT | TAGGCATTGTCATGGAGT |
| O65581 | Fructose-bisphosphate aldolase 5, cytosolic | TCACTTAGACATTCTCCGTTGC | CGAAGGCTTATCCGCTCC |
| Q00326 | Myrosinase | ACTTGGAATGGCTCTGCT | CCTTCTCATGCTGGGACT |
| Q42624 | Glutamine synthetase, chloroplastic | TTAGCGGCTTGTGGAGTC | GCAAAGGGTGCAGGGTAC |
| Q9LQL0 | Glycine cleavage system H protein 3, mitochondrial | GTTTGTCCACTGCGTTTA | CTGCTTCCGTCTTCATAG |
| P04778 | Chlorophyll a-b binding protein 1 | GAGATCGAGGAAGAGGTA | AATGAAACAATCGTCCAG |
| Q940Z5 | Phenolic glucoside malonyltransferase 1 | TGGCTTCTATGTTGACTC | TTCTTTCTGATGGCTTCC |

| **Gene Name** | **Forward (5'→3')** | **Reverse (5'→3')** |
| --- | --- | --- |
| *β-actin* | GACAGGATGCAGAAGGAGATTACT | TGATCCACATCTGCTGGAAGGT |
